# Cisplatin-Based Chemotherapy of Human Cancers

**Published:** 2019-04-08

**Authors:** Andrea Brown, Sanjay Kumar, Paul B Tchounwou

**Affiliations:** Cellomics and Toxicogenomics Research Laboratory, NIH/NIMHD-RCMI Center for Environmental Health, College of Science, Engineering and Technology, Jackson State University, 1400 Lynch Street, Box18750, Jackson, Mississippi, MS 39217, USA

**Keywords:** Cisplatin, Platinum-based dugs, Cancer chemotherapy, Human cancers

## Abstract

Cisplatin (cis-diammine-dichloro-platinum II) was initially discovered to prevent the growth of *Escherichia coli* and was further recognized for its anti-neoplastic and cytotoxic effects on cancer cells. Administered intravenously to humans, cisplatin is used as first-line chemotherapy treatment for patients diagnosed with various types of malignancies, such as leukemia, lymphomas, breast, testicular, ovarian, head and neck, and cervical cancers, and sarcomas. Once cisplatin enters the cell it exerts its cytotoxic effect by losing one chloride ligand, binding to DNA to form intra-strand DNA adducts, and inhibiting DNA synthesis and cell growth. The DNA lesions formed from cisplatin-induced DNA damage activate DNA repair response *via* NER (nuclear excision repair system) by halting cisplatin-induced cell death by activation of ATM (ataxia telangiectasia mutated) pathway. Although treatment has been shown to be effective, many patients experience relapse due to drug resistance. As a result, other platinum compounds such as oxaliplatin and carboplatin have since been used and have shown some levels of effectiveness. In this review, the clinical applications of cisplatin are discussed with a special emphasis on its use in cancer chemotherapy.

## Introduction

**Figure 1:**
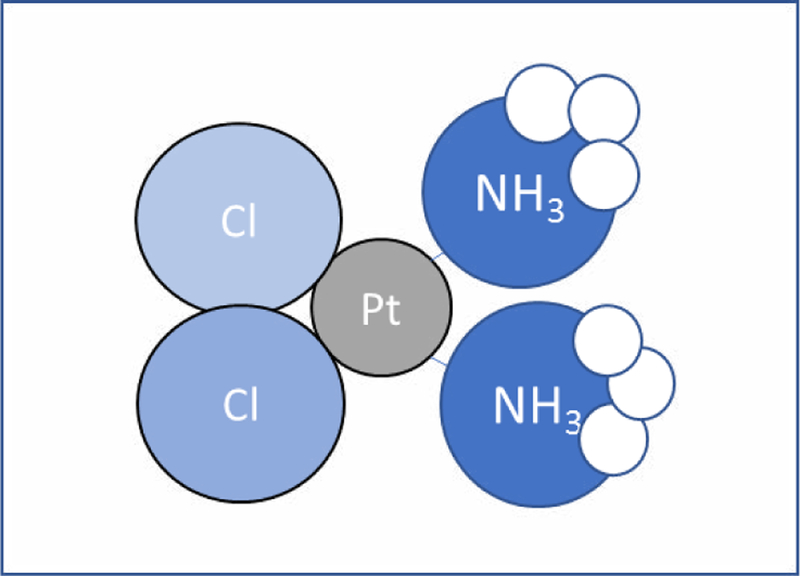
Molecular structure of cisplatin: This platinum compound is centered around two adjacent chloride ligands (on the left) and two amine groups (on the right).

Cisplatin (Cis-diammine-dichloro-platinum II) is an organometallic platinum compound that has two adjacent chlorine and amine atoms (Figure 1). Its anti-bacterial effects were first elucidated based on its inhibitory action on the growth of *Escherichia coli*. It was later discovered that cisplatin had potent anti-neoplastic effects on tumor cells [1]. Its clinical uses during the 1980s proved to be a breakthrough discovery in the establishment of successful cancer treatment drugs. Cisplatin, and other platinum-based compounds such as oxaliplatin, and carboplatin are still being used as first-line treatments for patients who have been diagnosed with various types of malignancies, such as lymphomas, breast, testicular, ovarian, head and neck, cervical, and sarcomas [1]. Seemingly, in the 1990s the rate of platinum-compound drug development was reduced. However, cisplatin and carboplatin remained the most stable and popular of platinum drugs in use. Although many other platinum analogues were tested in clinical studies, only cisplatin and carboplatin showed great benefits.

The cytotoxic mechanism of cisplatin is initiated by its interaction with DNA to form adducts; leading to apoptosis or programmed cell death [2]. Cisplatin was the first implementation of platinum-based chemotherapy introduced by Michele Peyrone in 1845 [3] and further tested in 1968 against multiple bacteria. The antineoplastic drug was administered intra-peritoneally to mice with traits of normal transplantable tumors such as sarcoma-180, at the dosage of 8 mg/kg; and surprisingly was found to be effective at marked tumor reversal [4]. In addition, due to the validity and reliability from *in vivo* tests conducted in the United Kingdom at the Chester Beatty Institute in London, cisplatin was further tested in clinical trials by the National Cancer Institute in the United States. In 1971, the initial implementation of cisplatin chemotherapy of cancer patients was done, and it was further approved by the United States Food and Drug Administration in 1978 [5]. Despite the success of cisplatin, the development of new platinum compounds represents a major focus intended to make cisplatin-based chemotherapy much safer for patients by minimizing adverse effects, nephrotoxicity, reoccurrence, and resistance [5].

Although many patients being administered cisplatin initially show a good reaction to its chemotherapy, some of them eventually relapse and develop resistance; leading to the reduction in its clinical usefulness. Evidence from tissue culture studies reveals that drug resistance may stem from epigenetic modifications at both cellular and molecular levels; including high levels of DNA damage repair (DDR), modifications in DNA methylation progress, uprooted and low regulation of mRNA expression levels, impairment in transcriptional regulation, and interference with apoptosis [6]. In the review, we provide a comprehensive overview of the medical applications of cisplatin in cancer chemotherapy.

## Molecular Mechanisms of Cisplatin Cytotoxicity

**Figure 2:**
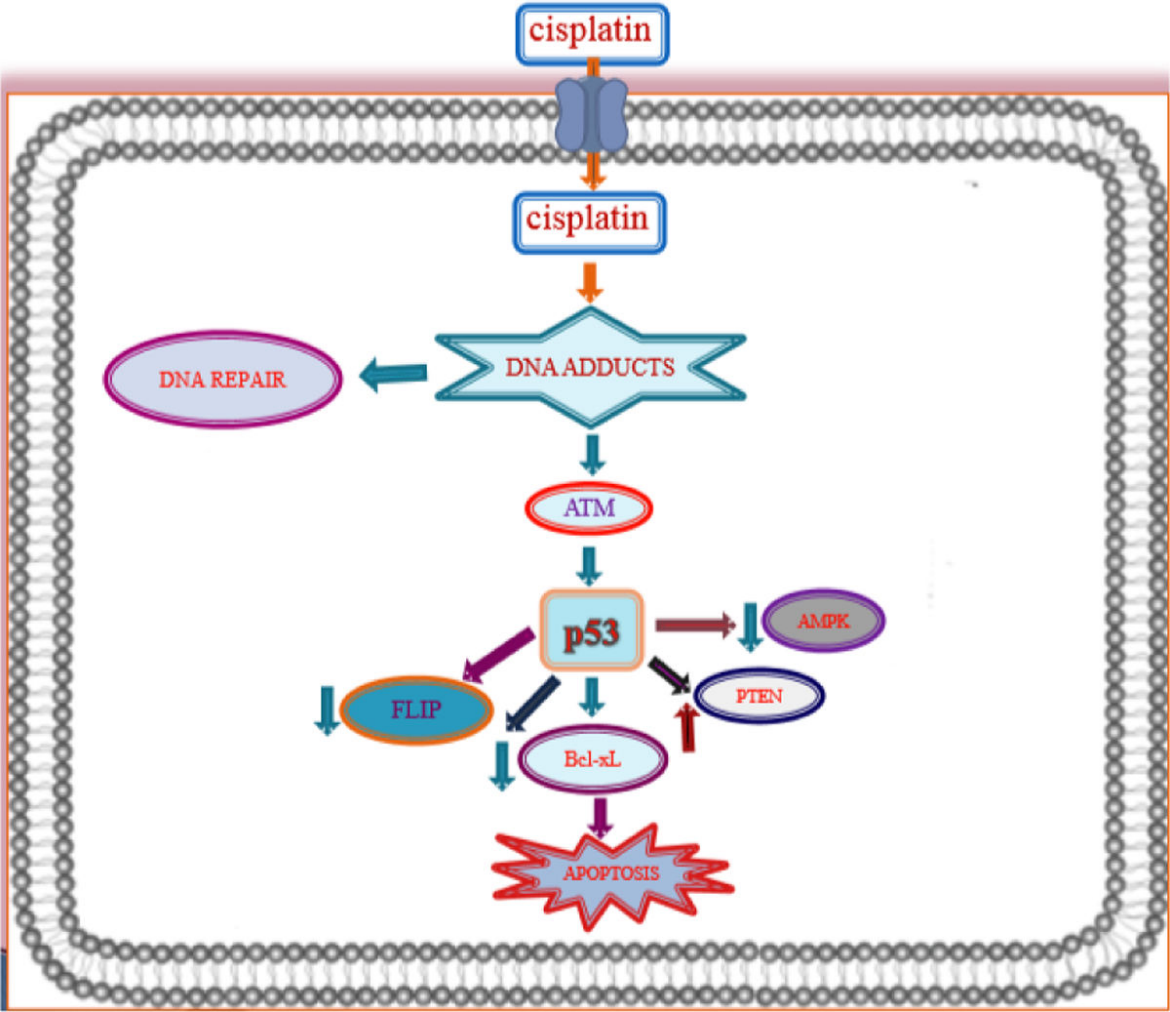
Overview of molecular mechanisms of cisplatin cytotoxicity: Cisplatin enters into cancer cells and interacts with DNA to form DNA adducts. It regulates protein kinase (ATM) and activates p53 leading to a series of signaling cascade and apoptosis in cancer cells.

Once cisplatin enters the cell it exerts its cytotoxic effect by losing one chloride ligand, binding to DNA to form intra-strand DNA adducts, and inhibiting DNA synthesis and cell growth. The DNA lesions formed from cisplatin-induced DNA damage activate DNA repair response *via* NER (nuclear excision repair system) by halting cisplatin-induced cell death by activation of ATM (ataxia telangiectasia mutated) pathway [7]. However, because cisplatin-induced DNA damage activate several signal transduction pathways that can facilitate or prevent apoptosis, studies have shown gene p53 is also associated in DNA damage and repair [8–11]. Moreover, once ATM is activated it maintains and phosphorylates tumor suppressor gene p53 which may induce transactivation of several genes inclusing p21 gene responsible for cell cycle growth arrest, DNA damage inducible gene 45 (GADD45) involved in DNA repair, and Bax to facilitate in apoptosis [12]. Subsequently, to aid in cisplatin-mediated apoptosis gene p53 has been shown to bind directly to Bax-xL to negate its anti-apoptotic activity [13] which decreases the effectiveness of FLICE-like inhibitory protein (FLIP) needed for p53 to activate cisplatin-mediated apoptosis [13,14]. Furthermore, the intra-strand lesions caused by cisplatin-induced DNA crosslinks activate the MMR, or mismatch repair system which promotes the activation of tyrosine kinase c-Abl in response to stress from DNA-damaging agents [15]. Upon activation of c-Abl, extracellular signals that maintain cell growth and sustenance such as JNK, p38 mitogen-activated protein kinase (MAPK) are activated to sustain tumor protein p73 resulting in programmed cell death [16]. Figure 2 provides an illustration of the cytotoxic mechanisms of cisplatin chemotherapy inside the cell membrane.

## Cisplatin and ovarian cancer

Eighty percent of patients who have ovarian cancer react to their first cell reduction surgery, superseded with some type of chemotherapy; with a combination of chemotherapeutic drugs cisplatin and paclitaxel or carboplatin and paclitaxel [17–19]. Consequently, 70% of patients who receive this type of treatment are at a disadvantage of ovarian cancer returning; with a higher percentage rate among patients with advanced levels of ovarian cancer [17–19]. Presently, cisplatin is one of the most powerful chemotherapeutic drugs used for the treatment of ovarian cancer; even though resistance is typical [20]. In ovarian germ cell cancer, the use of cisplatin brings about high response rates [21]. Since the malignancy in the ovaries is the most lethal of all gynecological cancers, knowledge of the molecular mechanisms of chemotherapy resistance in ovarian cancer is vital for the continuation of useful treatment and strategies to halt chemo-resistance [22].

## Cisplatin and testicular cancer

Testicular cancer, or malignant tumors that initiate in the testicles are most commonly found in men between ages 20–40 [23]. Treatment of testicular cancer by cisplatin and other platinum-based compounds has led to scientific breakthroughs, thus increasing the patients’ survival rate [24,25]. In contrast, many patients experience reoccurrence to such cancer due to platinum refusal; inducing late effects of palliative care forfeiting enhancement of life succeeding treatment. Since the mid-1970’s, when platinum-based, antineoplastic drugs, were first introduced, cisplatin-based chemotherapy has produced high endurance in testicular cancer (TC) patients, and likewise in patients with pervasive spread of the disease [26,27]. Dismally, not all testicular cancer patients achieve a complete cure; a portion of patients with pervasive malignancy do not attain lasting remission following their first treatment and later succumb to the disease [28].

## Cisplatin and head and neck cancer

Head and neck cancer (HNC) refers to malignant tumors stemming in the topmost aero-digestive tract; which include the lips, mouth, tongue, nose, throat, vocal cords, and part of the esophagus and windpipe [29]. HNC is ranked number eight among the most frequent types of cancers globally; with an incidence rate of more than 500,000 cases each year [30]. Interestingly, over 90% of head and neck tumors are classified as squamous cell carcinoma (or uncontrolled growth or malignant cells initiating in the epidermis) [29]. Undesirably, nearly 50% of these types of cancer are already at the progressive stage at the time of examination; and as a result, have to be treated using a number of approaches (i.e. radiotherapy, chemotherapy, and surgery) in order to preserve organs. Cisplatin is used as a first-line operative chemo-radiation therapy of HNC; usually being administered before or after surgery [31]. The most prevalent treatment regimen is called the Radiation Therapy Oncology Group RTOG) schedule; where cisplatin is administered in 100 mg/m^2^ on days 1, 22, 43 respectively, in consolidation with traditional radiotherapy [32]. By preference under the RTOG schedule, cisplatin can also be administered at 40 mg/m^2^ along with traditional or increased radiotherapy [33–35]. In addition, cisplatin in combination with known chemotherapeutic drugs docetaxel and fluorouracil have been deemed to have the highest effectiveness for induction treatment as compared to the combination of cisplatin and fluorouracil when treating locally advanced head and neck cancer [36,37].

## Cisplatin and esophageal cancer

Cisplatin is among the most efficient and extensively used anti-neoplastic agents clinically for treating patients diagnosed with esophageal cancer [38]. Esophageal cancer is ranked number nine among the most prevalent types of cancer and number six in mortality rates among all malignancies, globally; with greater than 80 % of all cases and deaths stemming from evolving nations [39]. Although removing part of or the whole esophagus is the first-line treatment in non-advanced stages; higher than 50% of patients are subject to cancer metastasis. Hence, chemotherapy is integral for palliative care [40,41]. Drug combination of cisplatin and fluorouracil has been used as a basic antidote for esophageal cancer patients as well as for those experiencing reoccurrence or at advanced stages [42]. Sadly, cisplatin treatment has some limitations most notably due to its refusal by cancer cells overtime. Also, the fundamental mechanisms of its refusal have yet to be determined [43]. Organic cation transporters (OTCs) are essential in the cellular response to cisplatin [44].

## Cisplatin and lung cancer

Lung cancer is found to be the leading cause of cancer mortalities globally. Its mortality rate is 36% per every 100,000 in China [45]. Additionally, non-small cell lung cancer (NSCLC) is responsible for almost all cancer-related deaths; particularly in patients in progressive stages III and IV of the disease [46,47]. Cis-diammine-dichloro-platinum (II) or cisplatin is regarded as the most popular anti-neoplastic drug to treat NSCLC [48]. However, cisplatin is also known to induce unfavorable side effects and drug resistance, especially after long-term exposure [48]. Combining cisplatin with other chemotherapeutic drugs such as paclitaxel, gemcitabine, docetaxel, or vinorelbine, represents a basic method for initial treatment of NSCLC [49]. Furthermore, strengthening the awareness of malignant cells to cisplatin in low doses is still an objective for the effectiveness of chemotherapy [50].

## Cisplatin and breast cancer

Breast Cancer (BC) is among the most prevalent types of cancer diagnosed in women; with majority of instances happening in women over the age of 60 [51]. Similar to the treatment of other malignancies, cisplatin is also used as the first-line agent for BC treatment [52]. Radiotherapy, surgery, and chemotherapy or any combination of these approaches has been used for BC treatment [51]. Carboplatin, another platinum-based, anti-neoplastic drug, has been used to treat breast cancer; however, cisplatin is still the most useful drug in healing BC [53]. According to the Gynecologic Oncology Group (GOG) the combination of cisplatin combined and doxorubicin has been considered as regular treatment based on Phase III clinical trials data [54]. Nevertheless, the widespread use of cisplatin as an effective anti-neoplastic drug has been limited due to side effects including nephrotoxicity, neurotoxicity, hepatotoxicity, and myelosuppression [55–58].

## Cisplatin and cervical cancer

Cervical cancer is ranked number two among the most prevalent malignancies and number three as the main cause of mortality in women in developing countries. Globally in 2012, its incidence and mortality cases were of 527, 600 and 265,700 respectively [59]. Cisplatin combined with pelvic-radiotherapy is the basic regimen for patients with increased risk factors and/or receiving surgery at premature stages [60,61]. Concurrent administration of cisplatin and paclitaxel is a common form of chemotherapy for advanced or reoccurring uterine cervical; according to a Phase III clinical trial by the Gynecologic Oncology Group [62]. The epidermal growth factor receptor (EGFR) may be a biomarker in cervical cancer because of its high expression ranging between 60–90%; and in some studies, has even been affiliated with lack of prognosis [63]. Moreover, such EGFR levels were high in cisplatin refusal malignancies versus cancers sensitive to cisplatin; mainly because cisplatin is prone to activating tyrosine phosphorylation in the EGFR, which is responsible for fixing DNA damage upon treatment with cisplatin [64,65].

## Cisplatin and gastric cancer

Gastric/stomach cancer (GC) is ranked number four among the most familiar types of cancer; with a yearly mortality of 738,000 cases worldwide [66]. Consequently, majority of patients with GC are not ascertained of their disease until it has metastasized due to inadequate capabilities of early detection [67]. Although chemotherapy is the standardized treatment for GC; one disadvantage is its multi-drug resistance (MDR) to several anti-neoplastic agents after being administered one chemotherapy drug [68]. Platinum-based drug cisplatin is known to treat GC and is popular at halting the development of malignant cells in humans [69]. Furthermore, analyzing cisplatin modes of action has pioneered the expansion of other combinations of chemotherapy drugs to attack an assortment of stable tumors resistant to cisplatin [70].

## Cisplatin and prostate cancer

Malignant tumor initiating in the male prostate or prostate cancer (PC) is considered to be the second common cancer in the world, and is responsible for 10% of all malignancies in men [71,72]. Cisplatin chemotherapy is first-line of treatment in conjunction with carboplatin and oxaliplatin because these platinum-based compounds interact with DNA to form DNA adducts in malignant cells, and induce programmed cell death or apoptosis [73–75]. In addition, refusal of cisplatin in PC3 cell lines is assumed to be due to the mutation and/or the nonoperation of p53 gene [76].

## Cisplatin and neuroblastoma

Cancer occurring in the sympathetic nervous system in infants and children is known as neuroblastoma [77]. Chemotherapeutic drugs such as cisplatin, carboplatin, vincristine, and etoposide are the primary drugs for chemotherapy of neuroblastoma. Reoccurrence of the disease along with drug refusal malignant cells are an interference of the advancement of curing patients with neuroblastoma [78]. It is presumed that resistance stems from epigenetic and several genetic modifications resulting in protein expression and abnormal RNA [79].

## Cisplatin and multiple myeloma

Multiple myeloma (MM), or cancer initiated in plasma cells found in bone marrow is still an incurable disease and despite the achievement of initial therapy; and so many patients with MM experience reoccurrence of the disease [80]. Research has pointed out that the general survival rate of MM patients is about nine months; with five months without reoccurrence [81]. Presently there is no basic form of treatment for multiple myeloma patients resistant to chemotherapeutic agents who do not meet the criteria for involvement of clinical trials [82]. However, the consolidation of dexamethasone, cyclophosphamide, etoposide, and cisplatin has shown effectiveness in treating MM patients [83].

## Cisplatin and melanoma

Malignant tumors in the cutaneous melanoma is the most destructive type of skin cancer. However, once the disease has metastasized toward the localized lymph nodes endurance rate is 29% and is further reduced to only 7% when the disease has spread towards major organs [84]. Anti-tumor agent cisplatin has been known to treat melanoma for the past 30 years; exerting its toxic effects by forming DNA adducts and damaging the DNA, leading to programmed cell death. On the contrary, the platinum-based chemotherapeutic drug is comparably resistant to melanoma, despite its efficacy towards other malignancies [85]. Some assumptions that may explain its chemo-refusal to cisplatin are errors in cellular-programmed death signaling, highly monitoring of DNA repair, and the interference of amassing agents from drug pumps [86].

## Cisplatin and mesothelioma

Malignant mesothelioma from tumor cells forming in the lining of the chest and abdomen is a rare, yet intrusive, type of cancer causing less than 1% of cancer mortality worldwide [87]. Cisplatin administered simultaneously with other chemotherapy drugs, or used as a single agent, is a standard treatment for malignant mesothelioma; even though its usefulness is narrow due to resistance [88]. Early detection of mesothelioma is poor; and on average patients survive only 1 year after diagnosis [89]. Radiotherapy and surgery are other procedures used in the treatment of mesothelioma; however, cisplatin chemotherapy is still highly favored [90]. Other possible combinations such as piroxicam and cisplatin have illustrated upgraded anti-tumor effects; heightening chances for survival in both *in vitro* and laboratory animal studies [91]. Such combination has also been reported to modulate cell-cycle progression, inducing programmed cell death or apoptosis [92]. Despite the emerging success of cisplatin and piroxicam, cisplatin use in mesothelioma treatment remains limited due to its toxicity.

## Cisplatin and leukemia

Leukemia or cancer in the blood is a well-known malignancy affecting a large number of people worldwide. Its incidence and mortality rates in children have been estimated to be 26% and 20%, respectively [93]. In the beginning stages, cellular distinction and function are widely managed; allowing productive therapy and death is minimal. However, without adequate treatment, the disease vastly progresses to lethal acute myeloid leukemia, or blastic phase; regrettably at this phase, the progressed disease is nearly incurable with modern drug therapy [94]. Since the approval of cisplatin by the Food and Drug Administration (FDA) in 1978; it has been an increasingly used anti-neoplastic agent in nearly 70% of patients as a component of treatment [95,96]. Although the implementation of platinum-based compounds has led to successful chemotherapy, its helpfulness in treating several malignancies has been diminished due to drug refusal and toxicity [97].

## Cisplatin and bladder cancer

Bladder cancer is ranked the highest in incidence rates of all cancers of the urinary system, in China [98]. Surgery and chemotherapy are the first-lines of treatment for bladder cancer. Cisplatin is known to be largely used in bladder cancer, showing potent anti-neoplastic effects [99]. However, some bladder cancer patients experience low sensitivity to cisplatin leading to drug resistance; altering its overall effectiveness of chemotherapy [100,101].

## Cisplatin and Hodgkin’s and non-Hodgkin’s lymphoma

Hodgkin’s lymphoma (HL) is a result of malignant tumors that initiate in the lymphatic system. Although its pathology is unfamiliar, the molecular and immunological complexity of HL suggests that these tumors arise from B-cells [102]. Minimum differentiation among children and adults in clinical and histological limitations has been explained [103]. Almost all HL patients experience successful treatment results with initial therapy, 30%−40% of HL patients in progressive stage are unsuccessful at full remission or experience reoccurrence [104,105]. In the United Kingdom suitable treatment for most HL patients upon relapse, include restorative chemotherapy and subsequent autologous stem-cell transplantation. Restorative regimens consist of high dose cisplatin and cytarabine, etoposide, and methyl-prednisolone; with response rates ranging between 60%−85% [106,107]. Similarly, to HL, the basic treatment for patients with non-Hodgkin’s Lymphoma (NHL) is high administration chemotherapy combined with autologous stem-cell transplant for initial treatment and for patients who experience reoccurrence of NHL [108].

## Conclusion

Cisplatin is a potent chemotherapeutic drug that has shown efficacy for the treatment of many malignancies including head and neck, ovarian, sarcomas, lymphoma, and prostate cancer. Once inside the cell, it exerts its cytotoxic effect by losing one chloride ligand, and forming DNA adducts resulting in cisplatin-induced DNA lesions. In response to this effect, several signaling transduction pathways are activated such as the mitogen-activation protein kinase pathways, DNA repair genes (Gadd45), p53 regulatory protein (mdm2), and several proteins that modulate apoptosis (bax, bcl-x1, bcl-2). Although treatment has been shown to be effective, many patients experience relapse due to drug resistance. As a result, other platinum compounds have since been used and have shown some levels of effectiveness. Cisplatin has also been administered in combination therapy as a method to reduce adverse side effects and resistance. Further research on cisplatin’s cytotoxic molecular mechanisms and synergistic effects with other cancer drugs is needed for the development of more effective anti-tumor drug treatment regimens.
